# Doxorubicin Dose-Dependent Impact on Physiological Balance—A Holistic Approach in a Rat Model

**DOI:** 10.3390/biology12071031

**Published:** 2023-07-22

**Authors:** Ana I. Afonso, Ângela Amaro-Leal, Filipa Machado, Isabel Rocha, Vera Geraldes

**Affiliations:** 1 Cardiovascular Centre of the University of Lisbon, 1649-028 Lisbon, Portugal; aiad@campus.ul.pt (A.I.A.);; 2 Egas Moniz School of Health and Science, 2829-511 Caparica, Portugal; 3 Institute of Physiology, Faculty of Medicine of the University of Lisbon, 1649-028 Lisbon, Portugal

**Keywords:** doxorubicin dosage, autonomic nervous system, HRV, physiological parameters

## Abstract

**Simple Summary:**

This study investigated how different doses of the chemotherapeutic drug doxorubicin affected physiological parameters and autonomic function in rats. A positive correlation was found between doxorubicin dose and blood pressure, heart rate, urinary norepinephrine, LF/HF ratio, and fibrotic heart area. We also found that higher doses of doxorubicin resulted in hypotension, bradycardia, and a decrease in the balance of autonomic activity (LF/HF), with 16 mg/kg being the threshold dose at which these changes became evident. These data suggest impaired control of cardiac function and highlight the importance of knowing the correct dosage of doxorubicin to minimize its adverse effects on the heart and improve treatment strategies for cancer patients.

**Abstract:**

Doxorubicin (DOX) is commonly used in several chemotherapies to treat various cancers, but it is known to cause cardiotoxicity and cardiac symptoms. Autonomic dysfunction is thought to contribute to the cardiotoxic effects of DOX, but the specific dose required to disrupt homeostatic processes is still unclear and is influenced by numerous factors. This study aimed to investigate how the DOX dosage affects autonomic function and physiological parameters, to elucidate the neurocardiac mechanisms underlying the observed cardiovascular side effects. Wistar rats were treated with DOX for four weeks and divided into three dosing groups: DOX8 (2 mg/kg/week), DOX16 (4 mg/kg/week), and DOX20 (5 mg/kg/week). A control group received NaCl 0.9% saline (1 mL/kg/week). In an acute experiment, we recorded blood pressure (BP), electrocardiogram, heart rate (HR), and respiratory rate (RF). Baroreflex gain and chemoreflex sensitivity were calculated, and cardiac tissue was analyzed with picrosirius histochemistry to measure collagen content. Our results showed that the LF/HF ratio, indicative of autonomic activity, was altered along with hypotension and bradycardia at a cumulative DOX dose threshold of 16 mg/kg. We observed a positive correlation between DOX dose and BP, HR, urinary norepinephrine, LF/HF ratio, and fibrotic heart area. Lower LF/HF ratios were associated with high DOX doses, reflecting drug-induced impairment of autonomic control of HR. This study provides valuable insights into the dose-dependent effects of DOX on physiological parameters and the development of cardiovascular dysfunction. These findings are critical, which is important for optimizing the management and therapeutic strategies for patients undergoing DOX-based chemotherapy.

## 1. Introduction

Doxorubicin (DOX) is a commonly used chemotherapeutic agent in clinical practice for various cancers, including leukemias, lymphomas, and solid tumors [1,2]. However, its clinical utility is often compromised by known side effects such as cardiotoxicity and cardiac symptoms, which may manifest years after initial exposure and lead to a poor prognosis [3]. Autonomic dysfunction has been implicated in the underlying mechanisms of DOX-related cardiotoxicity, with the sympathetic nervous system (SNS) playing an important role. However, the overall effects of DOX on the SNS are complex and multifactorial, depending on factors such as dose, treatment duration, and individual variability [4,5,6].

The specific dose of DOX required to affect the SNS remains uncertain and may depend on factors such as age, pre-existing cardiovascular disease, and concomitant use of drugs that may affect autonomic function. Clinical trials have attempted to assess the potential dose-dependent effects of DOX on homeostatic processes, often focusing on heart rate variability (HRV) as a non-invasive measure of autonomic function and assessing its effects on the autonomic nervous system [7,8,9].

Studies suggest that higher doses or longer treatment durations of DOX may lead to more pronounced effects on the SNS. Some studies have reported increased SNS activity following DOX administration [4,10,11,12,13]. Animal studies, such as that conducted by Moro et al., have shown that DOX treatment can increase sympathetic nerve activity in rats, which may contribute to the cardiotoxicity associated with this drug [14]. Similarly, in human studies, an increase in the low-frequency band (LF) has been observed after DOX treatment, indicating SNS activity [10,15,16,17]. Conversely, conflicting findings suggest that DOX may indirectly affect the SNS by damaging the myocardium, including neurotoxic effects on cardiac sympathetic neurons, revealing a previously unrecognized effect of DOX on cardiac autonomic regulation, which plays a crucial role in both cardiac physiology and pathology [14].

Several mechanisms shed light on how DOX negatively affects the autonomic nervous system and contributes to cardiovascular adverse effects. DOX can damage sympathetic neurons in the heart and affect the central nervous system, leading to mitochondrial degeneration, neuronal dysfunction, neurotoxicity, and changes in autonomic activity. These effects can occur through a variety of pathways, including oxidative stress, DNA damage, disruption of cellular processes, disruption of myocyte calcium balance, and inflammation [18,19,20,21,22,23].

Consequently, these mechanisms can affect autonomic reflexes, such as the baroreceptor and chemoreceptor reflexes, which are responsible for regulating blood pressure and ventilation, and they can cause inflammation, which activates the SNS and contributes to sympatho-excitation. Consequently, these changes can lead to cardiovascular side effects such as hypertension and cardiac arrhythmias. In addition, DOX may affect the release of neurotransmitters, including norepinephrine, an important neurotransmitter of the SNS. These cumulative changes can lead to altered autonomic activity and contribute to vascular dysfunction and significant changes in blood pressure [24,25].

However, several limitations and challenges hinder a clear understanding of the dose-dependent effects of DOX on autonomic function. These limitations include heterogeneity of study designs, lack of standardized measures, confounding factors such as age, sex, pre-existing cardiovascular disease and concomitant medications, and small sample sizes that may limit the statistical power to detect significant associations between DOX doses and impaired homeostatic processes [4].

Therefore, the present study aims to investigate the dose-dependent effects of DOX on physiological parameters and autonomic function in order to gain valuable insights into the neurocardiac mechanisms underlying the observed cardiovascular side effects. By investigating different DOX doses and their effects on autonomic function, our study aims to elucidate the relationship between DOX dosing and impaired homeostatic processes.

## 2. Materials and Methods

### 2.1. Animal Model Development

Female Wistar rats (n = 30), aged more than 12 weeks, were divided into four groups: a low DOX dosage (2 mg/kg/week, DOX8, n = 8), an intermediate DOX dosage (4 mg/kg/week, DOX16, n = 8), and a high DOX dosage (5 mg/kg/week, DOX20, n = 8) that received a weekly i.p. injection of DOX (doxorubicin hydrochloride Merck Life Science S.L., Barcelona, Spain, Ref D2975000) for 4 weeks [25,26,27]. An age-matched control group (CTL; n = 6; NaCl 0.9% saline solution; 1 mL/kg/week) without DOX treatment was used. The selection of DOX doses was guided by the aim of ensuring clinical relevance by incorporating a range of exposure levels. This includes a low cumulative DOX exposure (equivalent to approximately 48 mg/m^2^ in humans), an intermediate dose of DOX (equivalent to approximately 96 mg/m^2^), and a high dose of DOX (equivalent to approximately 120 mg/m^2^). The sample size calculation employed the resource equation method, using simple algebra to allocate a total of 30 animals across four groups. This approach ensured the attainment of an appropriate E value, aligning with the criteria outlined by Festing et al. [28].

Animals were housed in the animal facility of the Faculty of Medicine of the University of Lisbon, in a maximum number of 4 animals per cage, with controlled temperature (22 ± 1 °C) and humidity (50 ± 5%) and synchronized for 12/12 h light/dark cycle. Food (Mucedola, Italy) and tap water (Epal, Portugal) were provided ad libitum. All the experimental procedures were in accordance with the European Community legislation on animal experimentation (Directive 2010/63/EU) and were approved by the Ethical Committee of the Academic Centre of Lisbon (CAML). 

### 2.2. Physiological and Autonomic Evaluation

Animals were anesthetized with sodium pentobarbital (60 mg/kg, IP). The levels of anesthesia were maintained with a 20% solution (*v*/*v*) of the same anesthetic after assessing the withdrawal response. Rectal temperature was maintained through a homoeothermic blanket (Harvard Apparatus, Cambourne, UK). The trachea was cannulated below the larynx to record tracheal pressure. The femoral artery and vein were cannulated for blood pressure (BP) monitoring and injection of saline and drugs, respectively. The electrocardiogram (ECG) was recorded from subcutaneous electrodes placed into three limbs, and the heart rate was derived from the ECG recording (Neurology, Digitimer, Welwyn Garden City, UK).

The right carotid artery was cannulated, and chemoreceptors were stimulated by lobeline injection (0.2 mL, 25 µg/mL, Sigma, St. Louis, MO, USA). Baroreflexes were stimulated by phenylephrine injection (0.2 mL, 25 µg/mL, Sigma, St. Louis, MO, USA) in the femoral vein [29,30,31]. At the end of the above-mentioned acute experience, the animal was sacrificed with an overdose of anesthetic. The heart was then removed and placed in 4% paraformaldehyde (Sigma-Aldrich) at 4 °C for further histological studies.

### 2.3. Data Acquisition

BP, ECG, heart rate, and respiratory frequency were continuously recorded (PowerLab, ADInstruments, Colorado Springs, CO, USA) and acquired, amplified, and filtered at 1 kHz (Neurology, Digitimer, Welwyn Garden City, UK; PowerLab, ADInstruments, Colorado Springs, CO, USA). For basal autonomic evaluation, a baseline recording of 10 min was obtained. There was an interval of at least 3 min between each stimulation.

### 2.4. Data Analysis

#### 2.4.1. Baro- and Chemoreceptor Reflex Evaluation

The autonomic evaluation focused on the assessment of the overall autonomic tonus and the evaluation of baro- and chemoreceptor reflexes. The baroreceptor reflex gain (BRG) was quantified by calculating ΔHR/ΔBP (bpm/mmHg), upon phenylephrine provocation. The chemoreflex response was calculated through respiratory frequency (RF) derived from the tracheal pressure before and after stimulation with lobeline: ΔRF = RFstimulation-RFbasal. BP and HR were also evaluated.

#### 2.4.2. Analysis of BP and HR Variability

Cardiovascular variability is widely used for autonomic nervous system analysis [32,33]. For its implementation, Systolic BP and R-R interval data were analyzed (periods of 3 min) through wavelet transform using in-house software, Fisiosinal, to evaluate sympathetic (low-frequency band, LF, 0.15–0.6 Hz of SBP) and parasympathetic (high-frequency band, HF, 0.6–2.0 Hz of HR) activity over time [9,34]. The LF/HF ratio was computed as a measure of the autonomic tone [32,33].

### 2.5. Catecholamines ELISA

Animals were kept in metabolic cages for 24 h. Urine was collected and acidified with chloride acid 6M. Dopamine, noradrenaline, and adrenaline urinary concentrations were determined using commercially available ELISA kits for catecholamines (3 Catecholamines ELISA kit—Ref: BA-E-6600, ImmuSmol, Bordeaux, France). All ELISA samples and protocols were run in accordance with the manufacturer’s instructions.

### 2.6. Heart Histology

For histological examination, all samples were preserved in a 4% solution of paraformaldehyde until they were embedded in paraffin to allow the longitudinal sectioning of the entire heart into sections of 5 μm in thickness. After mounting these sections onto glass slides, they were stained with a picric acid–Sirius red solution (consisting of 0.1% Sirius red in saturated aqueous picric acid) to identify any cardiac fibrosis as stated elsewhere [35,36]. The stained sections were then examined through a colorimetric procedure, and digitized at a final 20× magnification using the image analysis and processing NanoZoomer SQ system. The digitalized images were then analyzed and picrosirius staining was quantified using a macro developed by the IMM-Bioimaging unit in accordance with Hadi et al. [37]. Picric acid–Sirius red staining showed the myocardial collagen stained in red [35,36].

### 2.7. Statistical Analyses

All data were analyzed using the software GraphPad Prism 9 (GraphPad Software Inc., Boston, MA, USA). The results are expressed as the mean ± standard error of the mean (SEM). Values of *p* < 0.05 or less were considered statistically significant. The normality distribution of the continuous variables was analyzed with the Kolmogorov–Smirnov test, and Levene’s test was used for the assessment of homogeneity of variance. Statistical differences were determined with one-way ANOVA Dunnett’s multiple comparison tests for comparison to the CTL group and unpaired *t*-tests for comparison inter-group (DOX8 vs. DOX16 vs. DOX20).

## 3. Results

### 3.1. Dose-Dependent Effects of DOX in Physiological and Autonomic Parameters

#### 3.1.1. DOX Treatment Leads to a Decrease in Blood Pressure and Heart Rate without Changes in Respiratory Frequency

**Figure 1:**
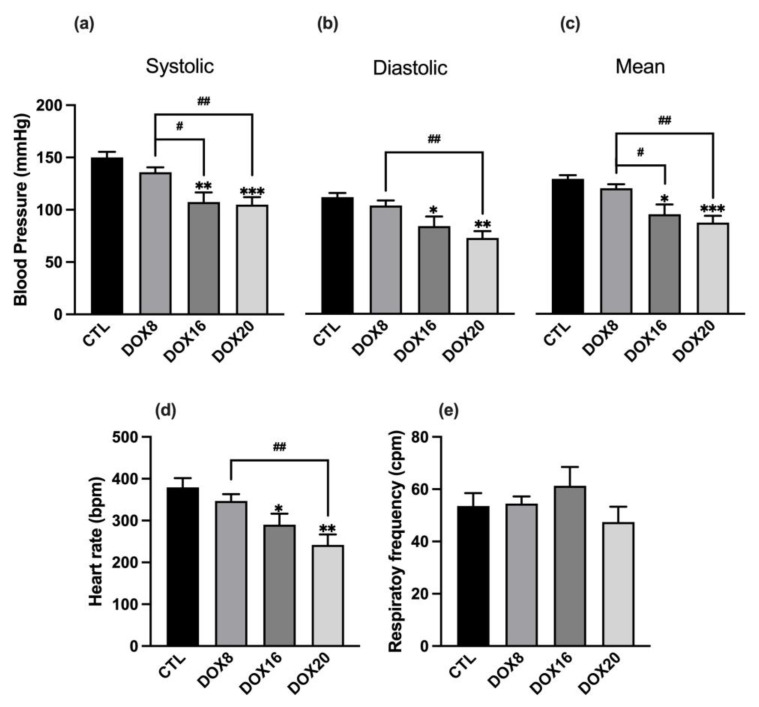
Dose-dependent effects of DOX in cardiovascular and respiratory parameters: (**a**) systolic, (**b**) diastolic, and (**c**) mean blood pressure. (**d**) Heart rate values were obtained from the electrocardiogram and (**e**) respiratory rate was calculated from tracheal pressure. Measurements were taken in all experimental groups: CTL, DOX8, DOX16, and DOX20. Values are mean ± SEM. * *p* < 0.05, ** *p* < 0.01, *** *p* < 0.001 for comparison to CTL group, one-way ANOVA, Dunnett’s multiple comparisons tests. # *p* < 0.05, ## *p* < 0.01 for comparison intergroup, unpaired Student *t*-test.

Cardiovascular and respiratory functions were evaluated in the acute experiment. The blood pressure data changes are depicted in Figure 1a–c, indicating a dependence on DOX dosage. Notably, the DOX20 dosage exhibits the most prominent hypotensive effect. Figure 1a–c shows that DOX16 and DOX20 groups presented a statistically significant decrease (*p* < 0.05) in systolic (sBP), diastolic (dBP), and mean blood pressure (mBP), when compared to the control group (CTL sBP: 150 ± 5.3 mmHg, dBP: 111 ± 3.9 mmHg and mBP: 129 ± 3.5 mmHg vs. DOX16 sBP: 107 ± 9.2 mmHg, dBP: 84 ± 9.2 mmHg, and mBP: 95 ± 9.3 mmHg, *p* < 0.05; and DOX20 sBP: 104.9 ± 7.2 mmHg, dBP: 73 ± 6.6 mmHg, mBP: 87 ± 6.6 mmHg, *p* < 0.05), without significant changes in DOX8 group (sBP: 136 ± 4.7 mmHg, dBP: 136 ± 4.7 mmHg, mBP: 136 ± 4.7 mmHg, *p* > 0.05). DOX16 and DOX20 showed a significant decrease in heart rate compared to the control group (379 ± 22 bpm vs. 290 ± 26 bpm and 242 ± 25 bpm, respectively, *p* < 0.05, Figure 1d). No changes in respiratory rate were observed (Figure 1e).

#### 3.1.2. DOX16 Caused Baroreflex and Chemoreceptor Reflexes Impairments

Baroreflex and chemoreflex are vital mechanisms that regulate autonomic reflexes in the body. Baroreceptors detect blood pressure changes and initiate appropriate responses, while chemoreceptors respond to oxygen and carbon dioxide variations [38]. Measuring the baroreflex provides insights into autonomic nervous system function, aiding in the diagnosis and monitoring of conditions such as hypertension, heart failure, and respiratory disorders [38,39,40]. Assessing these reflexes enhances our understanding of the body’s ability to regulate blood pressure and oxygen levels, contributing to improved knowledge of cardiovascular and respiratory health [38,39,40].

**Figure 2:**
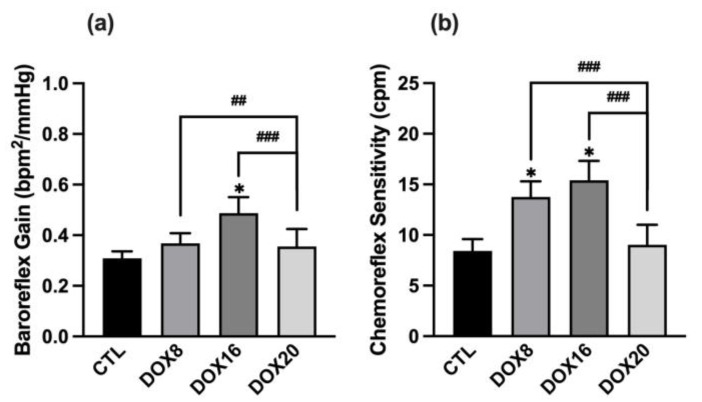
The histograms show the effect of different DOX dosages on baroreflex gain and chemoreflex variation. (**a**) BRG was calculated from the heart rate and blood pressure variation after phenylephrine stimulation. (**b**) Chemoreceptor reflex sensitivity is calculated from the variation in respiratory rate upon lobeline stimulation. Values are mean ± SEM. * *p* < 0.05 for comparison to CTL group, one-way ANOVA, Dunnett’s multiple comparisons tests. ## *p* < 0.01 and ### *p* < 0.001 for comparison intergroup, unpaired Student *t*-test.

Autonomic reflexes were pharmacologically provoked with phenylephrine, and the data are shown in Figure 2. Injection of phenylephrine triggered a progressive increase in the mean BP, which was accompanied by a progressive reduction in HR. No changes in the baroreceptor reflex gain (BRG) were found in all groups evaluated, except in the DOX16 group, which showed a significant increase in BRG when compared to the control group (Figure 2a: CTL 0.28 ± 0.02 bpm^2^/mmHg vs. DOX16 0.49 ± 0.06 bpm^2^/mmHg, *p* < 0.05), and DOX20 showed a decrease in BRG when compared to the DOX8 and DOX16 group (DOX20: 0.24 ± 0.3 bpm^2^/mmHg vs. DOX8: 0.37 ± 0.04 bpm^2^/mmHg, *p* < 0.05 vs. DOX16 0.49 ± 0.06 bpm^2^/mmHg, *p* < 0.001). Regarding chemoreceptor reflex sensitivity, a significant increase in the data was observed in DOX8 and DOX16 groups compared to the CTL and DOX20 groups (Figure 2b: DOX8 12.5 ± 1 bpm^2^/mmHg, DOX16 15.4 ± 1.9 bpm^2^/mmHg vs. CTL 8.4 ± 1.1 bpm^2^/mmHg, DOX20 7.2 ± 0.8 bpm^2^/mmHg, *p* < 0.05).

#### 3.1.3. Positive Correlation between DOX Dosage and Autonomic Nervous System Output

**Figure 3:**
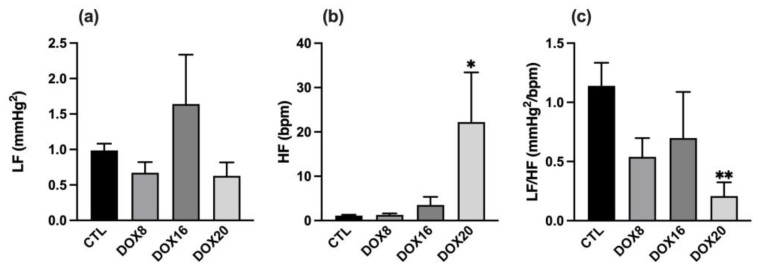
Dose-dependent DOX effect on sympathetic and parasympathetic tone. The sympathetic output was assessed by (**a**) LF, the parasympathetic output by (**b**) HF, and the balance between sympathetic and parasympathetic tone by (**c**) LF/HF ratio in all experimental groups: CTL, DOX8, DOX16, and DOX20. Values are mean ± SEM. * *p* < 0.05 and ** *p* < 0.01 for comparison to CTL group one-way ANOVA, Dunnett´s multiple comparisons tests.

DOX groups showed an overall decrease in cardiovascular autonomic outflow that is negatively correlated with the increase in DOX dosage. By using wavelet analysis applied to systolic BP and inter-pulse intervals, an observed decrease in the LF/HF ratio was correlated with the increasing dose of DOX (0.54 ± 0.16, 0.69 ± 0.39, 0.21 ± 0.12 mmHg^2^/bpm, *p* < 0.05). The parasympathetic output expressed by HF band power was from 1.13 ± 0.17 in CTL to 22.23 ± 11.18 bpm in DOX20, *p* = 0.0266 (Figure 3b). In contrast, the LF in all DOX groups remains unchanged (Figure 3a). The variations in the LF/HF, for each DOX group, are depicted in Figure 3c. High DOX dosage seems to associate with lower LF/HF balance, and this difference was statistically different when comparing the CTL group to the DOX20 group (1.14 ± 0.19 vs. 0.21 ± 0.12 mmHg^2^/bpm, *p* = 0.0054). No significant changes in the LF/HF balance were observed between DOX groups.

### 3.2. Changes in Urinary Catecholamines

**Figure 4:**
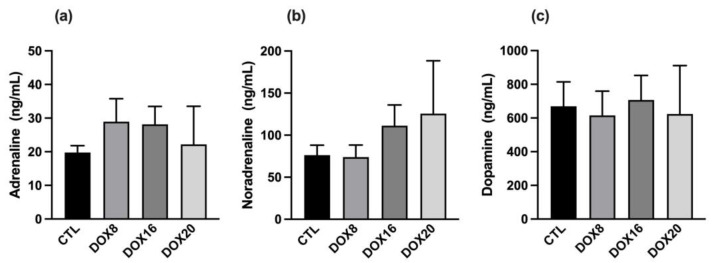
Dose-dependent effects of DOX on urinary adrenaline, noradrenaline, and dopamine. (**a**) The urinary concentration of adrenaline, (**b**) noradrenaline, and (**c**) dopamine were assessed in all experimental groups: CTL, DOX8, DOX16, and DOX20. Values are mean ± SEM. No statistical differences were observed.

Urinary adrenaline concentration was evaluated in all groups (Figure 4a), and no differences were found in CTL: 19.77 ± 2.044; DOX8: 28.91 ± 6.827; DOX16: 28.16 ± 5.302; DOX20: 22.20 ± 11.33 ng/mL). Additionally, no statistical difference was observed in urinary noradrenaline concentrations (Figure 4b); the data show a tendency for noradrenaline elevation with higher DOX dosages (CTL: 76.20 ± 11.89; DOX8: 73.96 ± 14.24; DOX16: 111.2 ± 24.75; DOX20: 125.6 ± 62.80 ng/mL). Urinary dopamine (Figure 4c) was similar between all groups evaluated (CTL: 669.5 ± 145.2; DOX8: 615.2 ± 144.8; DOX16: 707.0 ± 145.9; DOX20: 623.4 ± 287.6 ng/mL).

### 3.3. Increased Heart Fibrotic Area through Collagen Quantification with Higher DOX Dosages

**Figure 5:**
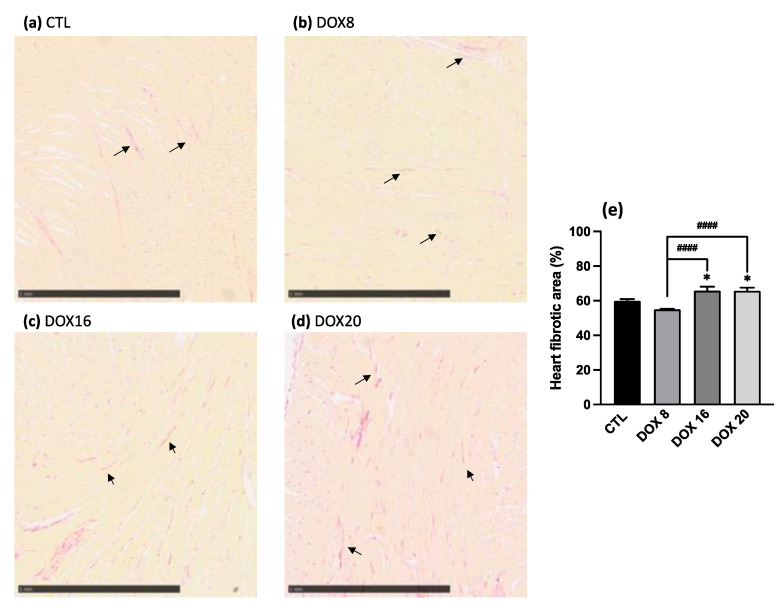
Representative images of collagen deposits in heart tissue in (**a**) CTL, (**b**) DOX8, (**c**) DOX16, and (**d**) DOX20 groups stained with picrosirius red. Arrows indicate the collagen fibers in the tissue. The scale bar provided in the image indicates that each mark corresponds to a length of 1 mm. The histograms (**e**) show the effect of different DOX dosages on the heart fibrotic area. The heart fibrotic area percentage was accessed by dividing the fibrotic area by the total area from all experimental groups: CTL, DOX8, DOX16, and DOX20. Values are mean ± SEM. * *p* < 0.05 for comparison to CTL group, one-way ANOVA, Dunnett´s multiple comparisons tests. #### *p* < 0.0001 for comparison intergroup, unpaired student *t*-test.

Heart tissue was stained for collagen quantification following the picrosirius histochemistry technique. Representative histology images are shown in Figure 5. The percentage of collagen fibrotic tissue was quantified, and data are represented in Figure 5e. An increase in cumulative DOX dosage leads to a consistent increase in heart fibrotic area when comparing the CTL group (59.9 ± 0.95) vs. DOX16 group (65.8 ± 2.36, *p* = 0.0394) and vs. DOX20 group (65.7 ± 1.88, *p* = 0.0208). The increase in heart fibrotic area is statistically different also between DOX8 (55.04 ± 0.25) vs. DOX16 (*p* < 0.0001) and vs. DOX20 group (*p* < 0.0001).

## 4. Discussion

The present study provides new insights into the dose-dependent effects of DOX on autonomic function and cardiovascular parameters. Our results present a positive correlation between the DOX dosage and physiological parameters, such as blood pressure, heart rate, urinary noradrenaline, LF/HF, and fibrotic heart area, and suggest that a cumulative dose of 16 mg/kg may serve as a threshold for the onset of adverse effects on blood pressure and heart rate. Interestingly, we observed a significant decrease in blood pressure and heart rate at higher doses of DOX, in contrast to the expected sympathetic excitation reported in most studies [41,42,43]. The discrepancy between our results and previous literature could be due to several factors, such as the cumulative dose used, the timing of the evaluation, the age of the animals, the experimental protocol, or the specific method used to assess autonomic function. In our study, wavelet analysis and the LF/HF ratio were used as measures of autonomic control [34,44].

Our study shows that at higher doses of DOX, the HF band increased rather than the LF band. This finding, associated with lower blood pressure and heart rate, suggests impaired autonomic regulation of heart rate caused by the drug. One possible mechanism associated with DOX-induced cardiotoxicity is chemoreceptor dysfunction. The chemoreceptors, located in the carotid and aortic bodies, are responsible for detecting changes in oxygen and carbon dioxide levels in the blood [45,46]. These receptors trigger reflexive responses to regulate respiration and cardiovascular function [46]. However, DOX treatment could lead to a dysfunction of the chemoreceptors and impair their ability to sense and respond to changes in blood gas levels. This dysfunction could contribute to the observed attenuation of the chemoreflex response due to reduced neuronal output, particularly seen at higher doses. Interactions with other signaling pathways that regulate autonomic function could also contribute to this finding. For example, DOX can induce oxidative stress and inflammation that can impair neuronal signaling within autonomic pathways [47]. This disruption of neuronal communication can lead to impaired autonomic regulation.

In addition, the effects of DOX on cardiac function and general health may indirectly influence the chemoreceptor reflex, potentially leading to an attenuated response. Understanding these potential mechanisms is important for DOX-induced cardiotoxicity, as the chemoreceptor reflex plays a critical role in maintaining cardiovascular homeostasis [44]. Impairments of the chemoreceptor reflex can disrupt autonomic regulation, leading to changes in blood pressure, heart rate, and respiration [46].

In the context of DOX-induced cardiotoxicity, a blunted chemoreflex response in the DOX20 group may contribute to the hypotension and decreased heart rate observed. In addition, the observed hypotensive effect may also be due to direct cardiac toxicity, as DOX is known to impair cardiac muscle function and disrupt calcium balance, leading to a decrease in cardiac inotropism [48]. This reduced pumping action of the heart could contribute to reduced vascular resistance and, consequently, lower blood pressure. In addition, DOX may cause endothelial dysfunction characterized by impaired vasodilation and increased vasoconstriction [49].

Endothelial cells play a critical role in regulating blood vessel tone and blood pressure. Disruption of endothelial function by DOX may result in decreased production of vasodilators such as nitric oxide and increased production of vasoconstrictors, leading to hypotension. Other factors such as direct vascular toxicity may also influence lower blood pressure in a complementary manner, especially at higher doses [50]. In fact, DOX has been shown to induce direct vascular toxicity, leading to endothelial cell damage and dysfunction, resulting in structural and functional changes in blood vessels, which in turn contributes to hypotension [50]. Of course, the reduced sympathetic activity observed in our study also contributed to the observed hypotension.

Indeed, DOX may have direct effects on the sympathetic nervous system, leading to a decreased release of norepinephrine, which can cause vasodilation and consequently hypotension [51]. In addition, the decrease in blood pressure and heart rate at higher doses of DOX may be related to an increase in baroreflex gain. This increase in baroreflex gain subsequently increases parasympathetic activity, which decreases heart rate. A sustained increase in baroreflex gain could potentially lead to a significant decrease in mean arterial pressure by suppressing the sympathetic nervous system.

Our results also showed an increase in fibrosis with the increase in DOX dose. These results are consistent with the literature [52,53,54]. Indeed, an association between DOX and cardiac fibrosis is well-established. DOX, as an anthracycline-containing chemotherapeutic agent, is associated with the development of cardiac fibrosis, which refers to the excessive deposition of collagen and other extracellular matrix components in cardiac tissue, leading to structural remodeling of the myocardium and impaired cardiac function.

The exact mechanisms by which DOX induces cardiac fibrosis are complex and not fully understood. However, several possible pathways have been identified, such as the formation of reactive oxygen species (ROS) leading to cardiac cell damage and fibroblast activation, the formation of reactive oxygen species causing oxidative stress in cardiac cells, the initiation of an inflammatory response in the heart with cytokines and growth factors release, promoting, therefore, fibroblast activation together with the activation of profibrotic pathways and endothelial dysfunction [18,55,56,57]. As our results show, the severity and extent of DOX-induced cardiac fibrosis depend on factors such as cumulative dose. The observed cardiac fibrosis may result in decreased myocardial contractility, impaired relaxation, and increased myocardial stiffness ultimately compromising cardiac function [58]. Accordingly, additional cardiac evaluation with echocardiographic parameters measurements will be essential to correlate the observed histological changes and cardiac dysfunction. Moreover, we are considering the implementation of other histological staining techniques to further validate and fortify our findings.

It is important to note that this study has limitations. An important aspect to consider in our study is the exclusive utilization of female animals, which may give rise to certain concerns. Nevertheless, it is crucial to acknowledge the existence of sex-related variations in the pharmacokinetics and pharmacodynamics of DOX [59,60]. The exclusive inclusion of female animals in our study raises the concern of potentially overlooking the influence of sex hormones on the outcomes of DOX treatment. To achieve a more comprehensive assessment of the drug’s effects, it would be beneficial to conduct a future study incorporating both male and female subjects, thereby accounting for the underlying hormonal disparities. Additionally, the observed effects are specific to the animal model used; further research is needed to confirm the results in humans. This approach will allow us to investigate the potential correlation between the observed histological changes and cardiac dysfunction. In addition, the study did not examine the long-term effects of DOX on autonomic function, which may provide additional insight into the development and progression of cardiotoxicity.

## 5. Conclusions

In conclusion, our study provides new insights into the dose-dependent effects of DOX on autonomic function and cardiovascular parameters. The observed decrease in blood pressure and heart rate, as well as the altered LF/HF ratio, indicate impaired autonomic control in response to DOX. The identified cumulative dose threshold of 16 mg/kg highlights the importance of dose monitoring and individualized treatment strategies. Our results confirm the association between DOX and increased fibrosis associated with oxidative stress, inflammation, and profibrotic metabolic pathways. Although our study has its limitations, further studies in humans are needed to investigate the long-term effects and clinical consequences of these dose-dependent autonomic changes.

Overall, our study improves our understanding of DOX-induced cardiotoxicity and highlights the need to monitor autonomic function and cardiovascular parameters in DOX-based chemotherapy. By improving our knowledge of these effects, treatment strategies can be optimized to achieve better outcomes for patients and will enable us to gain deeper insights into the mechanisms and long-term effects of DOX treatment.

## Data Availability

The data presented in this study are available on reasonable request from the corresponding author.

## References

[1] Awan P., Ingal K.S., Atasha N., Liskovic I., Boniface S. (1998). Doxorubicin-Induced Cardiomyopathy. N. Engl. J. Med..

[2] Young R.C., Ozolls R.F., Myers C.E. (1981). The Antracycline Antineoplastic Drugs. N. Engl. J. Med..

[3] Zamorano J.L., Lancellotti P., Rodriguez Muñoz D., Aboyans V., Asteggiano R., Galderisi M., Habib G., Lenihan D.J., Lip G.Y.H., Lyon A.R. (2016). 2016 ESC Position Paper on Cancer Treatments and Cardiovascular Toxicity Developed under the Auspices of the ESC Committee for Practice Guidelines. Eur. Heart J..

[4] Geraldes V., Caldeira E., Afonso A., Machado F., Amaro-Leal Â., Laranjo S., Rocha I. (2022). Cardiovascular Dysautonomia in Patients with Breast Cancer. Open Cardiovasc. Med. J..

[5] Norton N., Weil R.M., Advani P.P. (2021). Inter-individual Variation and Cardioprotection in Anthracycline-induced Heart Failure. J. Clin. Med..

[6] Von Hoff D.D., Layard M.W., Basa P., Hugh B.S., Davis L., Von Hoff A.L., Rozencweig M., Muggia F.M. (1979). Risk Factors for Doxorubicin-Lnduced Congestive Heart Failure. Ann. Intern. Med..

[7] Sztajzel J. (2004). Heart Rate Variability: A Noninvasive Electrocardiographic Method to Measure the Autonomic Nervous System. Swiss Med. Wkly..

[8] Huikuri H.V., Stein P.K. (2013). Heart Rate Variability in Risk Stratification of Cardiac Patients. Prog. Cardiovasc. Dis..

[9] Camm A.J., Malik M., Bigger T., Breithardt G., Cerutti S., Cohen R.J., Coumel P., Fallen E.L., Kennedy H.L., Kleiger R.E. (1996). Heart Rate Variability Standards of Measurement, Physiological Interpretation, and Clinical Use. Eur. Heart J..

[10] Potočnik N., Perše M., Cerar A., Injac R., Finderle Ž. (2017). Cardiac Autonomic Modulation Induced by Doxorubicin in a Rodent Model of Colorectal Cancer and the Influence of Fullerenol Pretreatment. PLoS ONE.

[11] El-Naggar A.E., El-Gowilly S.M., Sharabi F.M. (2018). Possible Ameliorative Effect of Ivabradine on the Autonomic and Left Ventricular Dysfunction Induced by Doxorubicin in Male Rats. J. Cardiovasc. Pharmacol..

[12] Nousiainen T., Vanninen E., Jantunen E., Remes J., Ritanen E., Vuolteenaho O., Hartikainen J. (2001). Neuroendocrine Changes during the Evolution of Doxorubicin-Induced Left Ventricular Dysfunction in Adult Lymphoma Patients. Clin. Sci..

[13] Tjeerdsma G., Meinardi M.T., Van Der Graaf W.T.A., Van Den Berg M.P., Mulder N.H., Crijns H.J.G.M., De Vries E.G.E., Van Veldhuisen D.J. (1999). Early Detection of Anthracycline Induced Cardiotoxicity in Asymptomatic Patients with Normal Left Ventricular Systolic Function: Autonomic versus Echocardiographic Variables. Heart.

[14] Moro N., Dokshokova L., Perumal Vanaja I., Prando V., Cnudde S.J.A., Di Bona A., Bariani R., Schirone L., Bauce B., Angelini A. (2022). Neurotoxic Effect of Doxorubicin Treatment on Cardiac Sympathetic Neurons. Int. J. Mol. Sci..

[15] Walsh D., Nelson K.A. (2002). Autonomic Nervous System Dysfunction in Advanced Cancer. Support. Care Cancer.

[16] Guimarães S.L.P.d.M.M., Brandão S.C.S., Andrade L.R., Maia R.J.C., Markman Filho B. (2015). Cardiac Sympathetic Hyperactivity after Chemotherapy: Early Sign of Cardiotoxicity?. Arq. Bras. Cardiol..

[17] Guo Y., Koshy S., Hui D., Palmer J.L., Shin K., Bozkurt M., Wamique Yusuf S. (2015). Prognostic Value of Heart Rate Variability in Patients with Cancer. J. Clin. Neurophysiol..

[18] Syukri A., Hatta M., Amir M., Rohman M.S., Mappangara I., Kaelan C., Wahyuni S., Bukhari A., Junita A.R., Primaguna M.R. (2022). Doxorubicin Induced Immune Abnormalities and Inflammatory Responses via HMGB1, HIF1-α, and VEGF Pathway in Progressive of Cardiovascular Damage. Ann. Med. Surg..

[19] Zhang S., Liu X., Bawa-Khalfe T., Lu L.S., Lyu Y.L., Liu L.F., Yeh E.T.H. (2012). Identification of the Molecular Basis of Doxorubicin-Induced Cardiotoxicity. Nat. Med..

[20] Shokoohinia Y., Hosseinzadeh L., Moieni-Arya M., Mostafaie A., Mohammadi-Motlagh H.R. (2014). Osthole Attenuates Doxorubicin-Induced Apoptosis in PC12 Cells through Inhibition of Mitochondrial Dysfunction and ROS Production. Biomed. Res. Int..

[21] Montaigne D., Hurt C., Neviere R. (2012). Mitochondria Death/Survival Signaling Pathways in Cardiotoxicity Induced by Anthracyclines and Anticancer-Targeted Therapies. Biochem. Res. Int..

[22] Osataphan N., Phrommintikul A., Chattipakorn S.C., Chattipakorn N. (2020). Effects of Doxorubicin-Induced Cardiotoxicity on Cardiac Mitochondrial Dynamics and Mitochondrial Function: Insights for Future Interventions. J. Cell. Mol. Med..

[23] Du J., Zhang A., Li J., Liu X., Wu S., Wang B., Wang Y., Jia H. (2021). Doxorubicin-Induced Cognitive Impairment: The Mechanistic Insights. Front. Oncol..

[24] Bosman M., Krüger D.N., Favere K., Wesley C.D., Neutel C.H.G., Van Asbroeck B., Diebels O.R., Faes B., Schenk T.J., Martinet W. (2021). Doxorubicin Impairs Smooth Muscle Cell Contraction: Novel Insights in Vascular Toxicity. Int. J. Mol. Sci..

[25] Robison T.W., Ciri S.N., Schiedt M., Parker H.R., Ishizaki G., Curry D.L. (1985). Effects of Intravenous Infusion of Doxorubicin on Blood Chemistry, Blood Pressure and Heart Rate in Rabbits?. J. Appl. Toxicol..

[26] Agostinucci K., Grant M.K.O., Melaku W., Nair C., Zordoky B.N. (2023). Exposure to Doxorubicin Modulates the Cardiac Response to Isoproterenol in Male and Female Mice. Pharmaceuticals.

[27] Podyacheva E.Y., Kushnareva E.A., Karpov A.A., Toropova Y.G. (2021). Analysis of Models of Doxorubicin-Induced Cardiomyopathy in Rats and Mice. A Modern View from the Perspective of the Pathophysiologist and the Clinician. Front. Pharmacol..

[28] Festing M.F.W., Altman D.G. (2002). Guidelines for the Design and Statistical Analysis of Experiments Using Laboratory Animals. ILAR J..

[29] Shvachiy L., Geraldes V., Amaro-Leal Â., Rocha I. (2020). Persistent Effects on Cardiorespiratory and Nervous Systems Induced by Long-Term Lead Exposure: Results from a Longitudinal Study. Neurotox. Res..

[30] Geraldes V., Carvalho M., Goncalves-Rosa N., Tavares C., Laranjo S., Rocha I. (2016). Lead Toxicity Promotes Autonomic Dysfunction with Increased Chemoreceptor Sensitivity. Neurotoxicology.

[31] Shvachiy L., Amaro-Leal Â., Outeiro T.F., Rocha I., Geraldes V. (2023). Intermittent Lead Exposure Induces Behavioral and Cardiovascular Alterations Associated with Neuroinflammation. Cells.

[32] Usui H., Nishida Y. (2017). The Very Low-Frequency Band of Heart Rate Variability Represents the Slow Recovery Component after a Mental Stress Task. PLoS ONE.

[33] Pichon A., Roulaud M., Antoine-Jonville S., De Bisschop C., Denjean A. (2006). Spectral Analysis of Heart Rate Variability: Interchangeability between Autoregressive Analysis and Fast Fourier Transform. J. Electrocardiol..

[34] Tavares C., Martins C., Superior I., Lisboa T., Laranjo P., Rocha S. Computational Tools for Assessing Cardiovascular Variability. Proceedings of the 1st Portuguese Biomedical Engineering Meeting.

[35] Junqueira L.C.U., Bignolas G., Brentani R.R. (1979). Picrosirius Staining plus Polarization Microscopy, a Specific Method for Collagen Detection in Tissue Sections. Histochem. J..

[36] Puchtler H., Waldrop F.S., Valentine L.S. (1973). Polarization Microscopic Studies of Connective Tissue Stained with Picro Sirius Red F3BA. Beiträge Pathol..

[37] Hadi A.M., Mouchaers K.T.B., Schalij I., Grunberg K., Meijer G.A., Vonk-Noordegraaf A., Van Der Laarse W.J., Beliën J.A.M. (2010). Rapid Quantification of Myocardial Fibrosis: A New Macro-Based Automated Analysis. Anal. Cell. Pathol..

[38] Halliwill J.R., Morgan B.J., Charkoudian N. (2003). Peripheral Chemoreflex and Baroreflex Interactions in Cardiovascular Regulation in Humans. J. Physiol..

[39] Benarroch E.E. (2008). The Arterial Baroreflex Functional Organization and Involvement in Neurologic Disease. Neurology.

[40] Schultz H.D., Li Y.L., Ding Y. (2007). Arterial Chemoreceptors and Sympathetic Nerve Activity: Implications for Hypertension and Heart Failure. Hypertension.

[41] Lončar-Turukalo T., Vasić M., Tasić T., Mijatović G., Glumac S., Bajić D., Japunžić-Žigon N. (2015). Heart Rate Dynamics in Doxorubicin-Induced Cardiomyopathy. Physiol. Meas..

[42] Buranakarl C., Kijtawornrat A., Chansaisakorn W., Trisiriroj M., Ngamdamrongkiat C., Taecholarn T., Sonpee J., Chanwathik W. (2014). Changes in Electrophysiology, Heart Rate Variability and Proteinuria in Clinical Dogs Treated with Doxorubicin Chemotherapy. Thai J. Vet. Med..

[43] Vasić M., Lončar-Turukalo T., Tasić T., Matić M., Glumac S., Bajić D., Popović B., Japundžić-Žigon N. (2019). Cardiovascular Variability and β-ARs Gene Expression at Two Stages of Doxorubicin–Induced Cardiomyopathy. Toxicol. Appl. Pharmacol..

[44] Laranjo S., Geraldes V., Oliveira M., Rocha I. (2017). Insights into the Background of Autonomic Medicine. Rev. Port. Cardiol..

[45] Schultz H.D., Sun S.-Y. (2000). Chemoreflex Function in Heart Failure. Heart Fail. Rev..

[46] Iturriaga R., Alcayaga J., Chapleau M.W., Somers V.K. (2021). Carotid Body Chemoreceptors: Physiology, Pathology, and Implications for Health and Disease. Physiol. Rev..

[47] Shi S., Chen Y., Luo Z., Nie G., Dai Y. (2023). Role of Oxidative Stress and Inflammation-Related Signaling Pathways in Doxorubicin-Induced Cardiomyopathy. Cell Commun. Signal..

[48] Octavia Y., Tocchetti C.G., Gabrielson K.L., Janssens S., Crijns H.J., Moens A.L. (2012). Doxorubicin-Induced Cardiomyopathy: From Molecular Mechanisms to Therapeutic Strategies. J. Mol. Cell. Cardiol..

[49] Menna P., Salvatorelli E., Minotti G. (2008). Cardiotoxicity of Antitumor Drugs. Chem. Res. Toxicol..

[50] Lenneman C.G., Sawyer D.B. (2016). Cardio-Oncology: An Update on Cardiotoxicity of Cancer-Related Treatment. Circ. Res..

[51] Medeiros-Lima D.J.M., Carvalho J.J., Tibirica E., Borges J.P., Matsuura C. (2019). Time Course of Cardiomyopathy Induced by Doxorubicin in Rats. Pharmacol. Rep..

[52] Tao H., Yang J.J., Shi K.H., Li J. (2016). Wnt Signaling Pathway in Cardiac Fibrosis: New Insights and Directions. Metabolism.

[53] Packard R.R.S. (2022). Cardiac Fibrosis in Oncologic Therapies. Curr. Opin. Physiol..

[54] Cappetta D., Rossi F., Piegari E., Quaini F., Berrino L., Urbanek K., De Angelis A. (2018). Doxorubicin Targets Multiple Players: A New View of an Old Problem. Pharmacol. Res..

[55] Rababa’h A.M., Guillory A.N., Mustafa R., Hijjawi T. (2018). Oxidative Stress and Cardiac Remodeling: An Updated Edge. Curr. Cardiol. Rev..

[56] Prud’homme G.J. (2007). Pathobiology of Transforming Growth Factor β in Cancer, Fibrosis and Immunologic Disease, and Therapeutic Considerations. Lab. Investig..

[57] Kalyanaraman B., Joseph J., Kalivendi S., Wang S., Konorev E., Kotamraju S. (2002). Doxorubicin-Induced Apoptosis: Implications in Cardiotoxicity. Mol. Cell. Biochem..

[58] Kang J., Cai L., Kang Y.J. (2001). Oxidative Stress and Diabetic Cardiomyopathy Oxidative Stress and Diabetic Cardiomyopathy A Brief Review. Cardiovasc. Toxicol..

[59] Soldin O.P., Mattison D.R. (2009). Sex Differences in Pharmacokinetics and Pharmacodynamics. Clin. Pharmacokinet..

[60] Liu Z., Martin J., Orme L., Seddon B., Desai J., Nicholls W., Thomson D., Porter D., McCowage G., Underhill C. (2018). Gender Differences in Doxorubicin Pharmacology for Subjects with Chemosensitive Cancers of Young Adulthood. Cancer Chemother. Pharmacol..

